# Hydrophobic Drug/Toxin Binding Sites in Voltage-Dependent K^+^ and Na^+^ Channels

**DOI:** 10.3389/fphar.2020.00735

**Published:** 2020-05-15

**Authors:** Kenny M. Van Theemsche, Dieter V. Van de Sande, Dirk J. Snyders, Alain J. Labro

**Affiliations:** Laboratory of Molecular, Cellular, and Network Excitability, University of Antwerp, Antwerp, Belgium

**Keywords:** hydrophobic binding sites, voltage-gated potassium channels, voltage-gated sodium channels, channel fenestrations, lipophilic compounds

## Abstract

In the Na_v_ channel family the lipophilic drugs/toxins binding sites and the presence of fenestrations in the channel pore wall are well defined and categorized. No such classification exists in the much larger K_v_ channel family, although certain lipophilic compounds seem to deviate from binding to well-known hydrophilic binding sites. By mapping different compound binding sites onto 3D structures of Kv channels, there appear to be three distinct lipid-exposed binding sites preserved in K_v_ channels: the front and back side of the pore domain, and S2-S3/S3-S4 clefts. One or a combination of these sites is most likely the orthologous equivalent of neurotoxin site 5 in Na_v_ channels. This review describes the different lipophilic binding sites and location of pore wall fenestrations within the K_v_ channel family and compares it to the knowledge of Na_v_ channels.

## Introduction

Voltage-gated ion channels are transmembrane proteins that are selectively permeable to physiological important ions such as Na^+^, K^+^, Ca^2+^, and Cl^-^. Under influence of the membrane potential (Vm) these channels change their conductance. In the conductive open (or activated) state, ions flow down their electrochemical gradient through the channel pore. The flux of these ions elicits an electrical current that directly influences the Vm. For voltage-gated sodium and potassium channels (Na_v_ and K_v_), the main focus of this review, the Vm will shift towards the ion's equilibrium potential, which under normal conditions is depolarizing and repolarizing, respectively (Hille, 2001). Although Na_v_ and K_v_ channels differ in selectivity from one another, their structure is quite similar. However, a main difference is that Na_v_ channels are characterized by one large α-subunit containing four recognizable domains (DI–IV), whereas K_v_ channels are formed by tetramerization of four individual α-subunits. In both cases, these four entities comprise six transmembrane segments (S1–S6), which are divided into a voltage sensing domain (VSD, S1–S4) and a pore-forming domain (PD, S5–S6) that are connected by the S4–S5 linker. The four PDs assemble into the ion permeation pathway (or pore) that is surrounded by four VSDs. In the non-conductive closed (or deactivated) state, ion permeation is prevented by the intracellular activation gate, located at the point where the four S6 helices cross. The aperture-like opening and closure of this gate is controlled by the VSD (Doyle et al., 1998; Bavro et al., 2012; Labro and Snyders, 2012; Lenaeus et al., 2017). The main component of the VSD is the S4 segment that physically moves in response to a change in Vm, due to the presence of positively charged residues (arginine and lysine) that detect changes in the membrane electric field (Bezanilla, 2008). The S4–S5 linker is a component of the electro-mechanical coupling that translates S4 movements into opening or closing of the activation gate (Bezanilla, 2008; Blunck and Batulan, 2012). After opening, fast inactivation occurs in Na_v_ and in some K_v_ channels, which is caused by the physical occlusion of the pore by an inactivation particle. For Na_v_ channels this inactivation particle is the linker between DIII and DIV, while in K_v_ channels it is located at the N-terminus of each subunit, hence termed N-type inactivation (Hoshi et al., 1990; West et al., 1992; Armstrong and Hollingworth, 2018). Alternatively, K_v_ channel inactivation can occur *via* the slower C- or U-type inactivation mechanism that makes the channels non or less conductive (Hoshi et al., 1991; Klemic et al., 1998; Cuello et al., 2010).

Binding of drugs/toxins to Na_v_ and K_v_ channels may alter the activation, deactivation, and/or inactivation process(es), which may cause or alleviate aberrant electrical excitability. Therefore, knowledge about the different binding sites is key for drug development and pharmacovigilance. The binding sites for these drugs/toxins are well defined and categorized within the Na_v_ channel family, as opposed to the much larger K_v_ channel family. Most binding sites are enveloped by water, locating either inside or outside the channel's pore. However, some compounds bind to a site(s) that does not fit any of the hydrophilic binding sites. For instance, brevetoxins and ciguatoxins bind to a conserved hydrophobic site within the Na_v_ channel family, termed neurotoxin site 5 (Catterall and Risk, 1981; Cestele and Catterall, 2000). For the K_v_ channel family no such site has been described, but certain compounds have been shown to deviate from binding to hydrophilic binding sites like; retigabine, gambierol, psora-4, polyunsaturated fatty acids (PUFAs), ICA-compounds (Vennekamp et al., 2004; Kopljar et al., 2009; Lange et al., 2009; Borjesson and Elinder, 2011). It is notable that these are rather lipophilic compounds and there has been, and still is, a growing interest in such compounds for their use in treating neurological disorders (e.g., as anti-convulsant).

So, is there a unifying picture of the lipid exposed/accessible drug/toxin binding sites within the large K_v_ channel family and even between K_v_ and Na_v_ channels? Several lipophilic binding sites have been described in different K_v_ channels, while in fact some may converge to just one binding region preserved between Kv channel (sub)families. In this review, the well-documented Na_v_ lipophilic binding sites, neurotoxin site 2, site 5, and the access to the local anaesthetic (LA) binding site within the pore through fenestrations is compared to what has been reported for K_v_ channels.

## Voltage-Gated Sodium Channels

The Na_v_ channel family contains nine isoforms (Na_v_1.1 to Na_v_1.9) that display a high sequence homology, especially within the transmembrane segments (Marban et al., 1998; Ahern et al., 2016). This facilitated the categorization of drug/toxin binding sites within the Na_v_ channel family. Over the past decades a detailed picture emerged on where compounds bind within these channels and resulted in a well-documented classification of seven different sites (site 1 to 7) and a LA binding site (Stevens et al., 2011; De Lera Ruiz and Kraus, 2015). As the focus of this review is on the binding sites that involve lipid soluble and/or transmembrane binding compounds only binding site 5 and site 2 will be briefly discussed. The LA binding site is also mentioned as some compounds can reach their binding site *via* hydrophobic fenestrations in the pore wall of the channel protein. To maintain an orderly overview, all Na_v_ residues are numbered according to the Na_v_1.4 channel when possible. In case the sequence could not be aligned, as for bacterial Na_v_ structures, it will be noted and the original numbering is maintained.

### The Closed State Accessible LA Binding Site: Pore-Accessibility Through Channel Fenestrations

**Figure 1:**
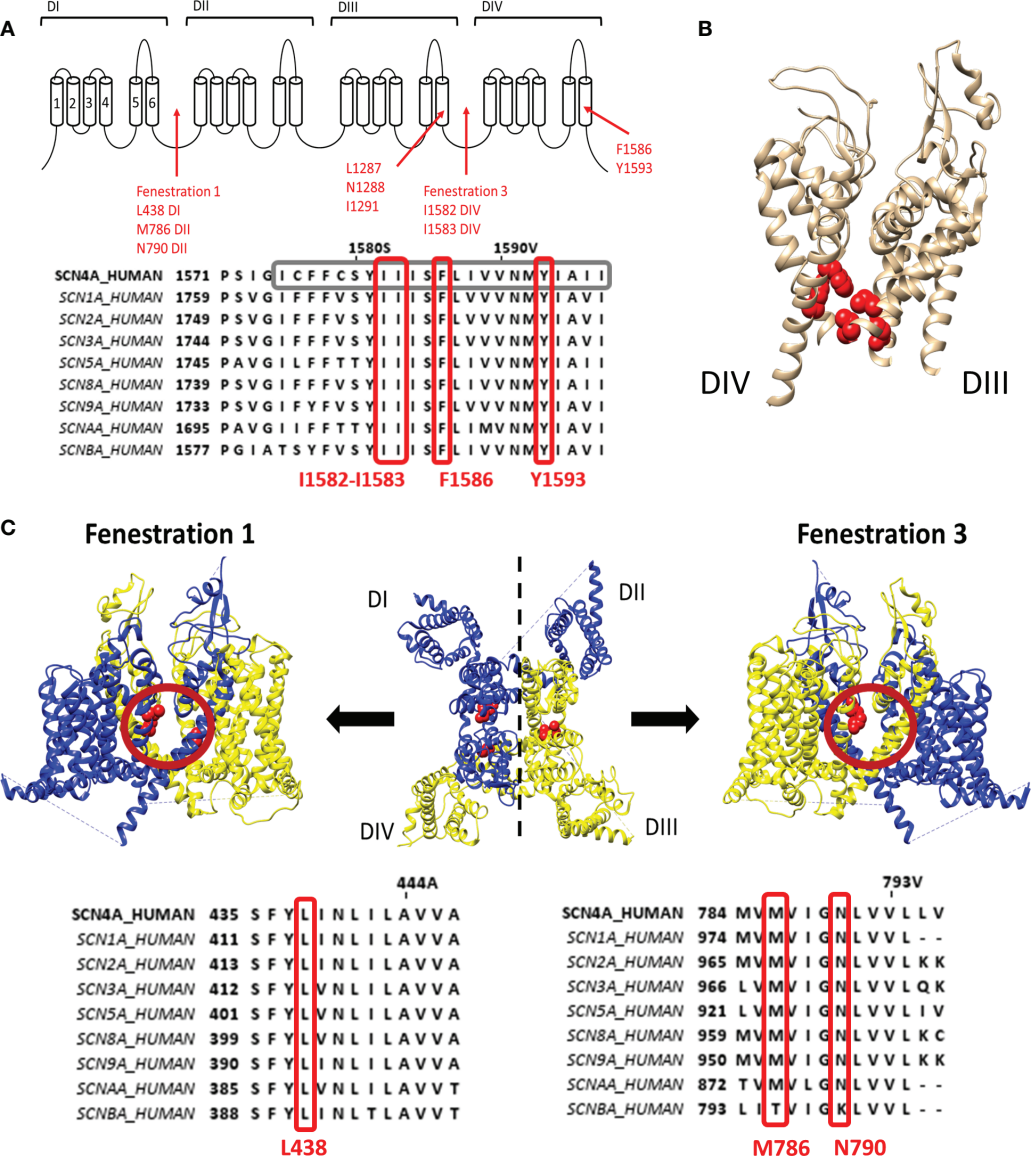
Representation of the local anaesthetic (LA) binding site and the hydrophobic access paths or fenestrations. **(A)** Schematic representation of the Na_v_ channel topology which contains four domains (DI to DIV) each consisting out of six transmembrane segments (S1–S6). Regions and location of residues important for LA binding are indicated with red arrows. Fenestrations that are sufficiently large to allow passage of LA compound are between DI–DII (fenestration 1) and DIII–DIV (fenestration 3) with the bottleneck residues listed below. This schematic representation is then followed by an alignment of Na_v_1.4 DIV with the other human isoforms, with marked in grey S6 and marked in red well conserved residues for LA binding and the fenestration bottleneck (I1582–I1583). Mutation of I1582 can also create a pathway connecting the inner pore with the extracellular environment. **(B)** 3D structure of the Na_v_1.4 channel representing the S5–S6 segments of DIII and DIV. In red are the residues, forming the LA binding site, visualized which are listed in panel A, clearly marking the inner pore LA binding site. **(C)** In the middle a top view of the 3D structure of the Na_v_1.4 channel is shown with in blue the DI–DII domains and in yellow the DIII-DIV domains. Fenestration 1 locates between DI and DII a side view of it shown on the left. A side view of fenestration 3 is shown on the right. Both fenestrations are highlighted by a red circle and the residues responsible for creating the bottleneck of the fenestration are represented in red. Below are the alignments of Na_v_1.4 with the other isoforms. On the left the bottleneck residue of DI is marked in red, on the right the bottleneck residues of DII. Amino acid sequence alignment and 3D structures are visualized using Jalview and chimera software, respectively (Pettersen et al., 2004; Waterhouse et al., 2009).

LA compounds and anti-arrhythmic drugs inhibit Na_v_ channels by occlusion of the pore. Most LA compounds have a similar structure consisting of a tertiary hydrophilic amine domain (head) linked with an aromatic hydrophobic ring domain, with a total length of 10–15Å (Courtney, 1988). Three types of block can be observed. First type is the use dependent open state block, or high affinity block, which occurs after channel opening and LA compounds enter the pore *via* the intracellular side (Grant et al., 1989; Benz and Kohlhardt, 1991; Gingrich et al., 1993). The second type is flicker block, or fast block, which is only observed when the channel's inactivation process is modulated. For example, by other compounds such as batrachotoxin (BTX) (CAS No.:23509-16-2) that binds at site 2 (Cahalan, 1978; Uehara and Moczydlowski, 1986; Zamponi et al., 1993). The third and least common type is the resting or hydrophilic block that establishes when the channel is closed. LA compounds are thought to enter the ion conductive pore and find their binding site trough fenestrations in the lipid exposed part of the PD (Gamal El-Din et al., 2018). LA compounds are protonated but for crossing the cell membrane they need to be deprotonated. This is possible when their pKA value is close to the physiological pH values in the extracellular environment. After traversing the cell membrane the compounds are protonated again to exert their pharmacological effect (Hille, 1977). As the binding site is located within the pore, block occurs with the highest affinity when the channel is opened or used, while applying frequent channel activating stimuli. Two residues within the S6 of domain four (DIV-S6) have been identified to be important for LA binding/modulation, namely, F1586 and Y1593, (Na_v_1.4 numbering). F1586 probably binds to the alkylamino head, while Y1593 interacts with the aromatic ring structure (Ragsdale et al., 1994; Yarov-Yarovoy et al., 2002) (**Figures 1A, B**). As these residues reside within the pore and certain compounds display closed state block, this requires a hydrophobic pathway to the binding site when the channel gate is closed (Hille, 1977).

These pathways, or fenestrations, in the lipid exposed part of the PD were first observed in the crystal structure of bacterial Na_v_ channels such as Na_v_Ab (Payandeh et al., 2011; Payandeh et al., 2012), Na_v_Ms (Mccusker et al., 2012) and Na_v_Rh (Zhang et al., 2012). It should be noted that bacterial Na_v_ channels are constructed out of four separate α-subunits instead of one large subunit. For the bacterial Na_v_ channels, four fenestrations are observed between the different domains where the radius of the fenestration varies from 0.8Å minimally to 2.59-2.83Å (Kaczmarski and Corry, 2014). Nonetheless, these fenestrations are wide enough for small LA compounds and anti-arrhythmic drugs to pass. The narrowest point in the fenestration, termed “bottleneck”, is created by the amino acid residues M174, T175, F203, T206, and M209 (Na_v_Ab numbering), with F203 being the most important residue (**Figure 1C**). These amino acids will sterically hinder the passage of compounds through the fenestration. Mutation of F203 to an alanine increased the size of the fenestration allowing easier access of flecainide (CAS No.:54143-55-4, polar surface area=59.6Å) to its binding site within the pore, with as result an increased tonic, closed state, block (Gamal El-Din et al., 2018). The mutation did not affect lidocaine block as this compound is smaller (polar surface area=32.3Å) and can easily traverse the wild type fenestration (Courtney, 1988). The F203W mutation on the other hand reduces fenestration size and consequently the access of both lidocaine and flecainide is reduced, decreasing tonic block (Gamal El-Din et al., 2018). Computational modelling using the Na_v_1.4 3D structure resulted in the observation of four fenestrations just as in bacterial Na_v_ channels, with the exception that the size of two out of the four fenestrations seemed inadequate for compound access, namely the fenestration constructed by DII–DIII and DIV–DI (Kaczmarski and Corry, 2014). Fenestrations in between DI-DII and DIII-DIV seemed sufficiently wide for compounds to cross (**Figure 1C**). The bottleneck of these fenestrations is formed by residues N790, L438, and M786 for the fenestration between DI-DII and by I1582, I1583, and F1586 (if rotated) for the fenestration between DIII-DIV. These fenestrations lining residues, are well conserved in the different Na_v_ channel isoforms. Mutating DIV-S6 residue I1582 appeared to create an extra pore access pathway (Ragsdale et al., 1994) and allowed the external blocker QX314 (CAS No.:24003-58-5, polar surface area: 29.1Å), which is a charged LA compound at physiological pH and therefore not able to traverse the membrane, to access the LA binding site when added in the extracellular environment (Sunami et al., 2001).

Apart from LAs that have a distinct binding site, sevoflurane (CAS No.: 28523-86-6), an inhalational anaesthetic, has a more complex binding profile. Sevoflurane binding results in a decrease of the peak sodium current, a hyperpolarised shift in the voltage dependence of inactivation and a slowing of the recovery from inactivation (Horishita et al., 2008; Ouyang et al., 2009). Within the bacterial sodium channel NaChBac, binding regions have been located at the pore region, selectivity filter, and the S4–S5 linker/S6 interface (Ouyang et al., 2007; Raju et al., 2013). MD simulations suggested that the binding of sevoflurane to the selectivity filter and the S4–S5 linker occurs mainly when the channel is in the activated/open state, while in the closed state the channel gate and the VSD are targeted (Barber et al., 2014). A state independent binding site is possibly the central cavity that is accessed by the fenestrations. Residues T220 and F227 (NavChBac numbering) are proposed to be responsible for sevoflurane binding in the central cavity, which are the homologue residues for LA binding in Nav1.4 (F1764, Y1771).

### Binding Site 2 Compounds, Though Being Lipophilic, Bind to the Inner Pore

Compounds binding at site 2 of the Na_v_ channel include batrachotoxin (Daly et al., 1965; Huang et al., 1982), grayanotoxin (Yuki et al., 2001; Jansen et al., 2012), CAS No.: 54781-61-2), and alkaloids from plant such as veratridine (Ulbricht, 1969; Sutro, 1986), CAS No.:71-62-5), aconitine and mesaconitine (Herzog et al., 1964; Friese et al., 1997) (CAS No.: 302-27-2). All these compounds are lipid soluble and need to traverse the cell membrane before influencing the channel in a use-dependent manner, comparable to the action of LA compounds (Herzog et al., 1964; Catterall, 1980; Huang et al., 1982; Dubois et al., 1983; Sutro, 1986; Barnes and Hille, 1988; Ameri et al., 1996; Yuki et al., 2001; Wang and Wang, 2003). Their effects can be a combination of: (1) a hyperpolarised shift of the voltage dependence of activation, (2) inhibition of the fast inactivation process, leading to persistent sodium currents, (3) decrease in ion conductance, and/or (4) decrease of the Na^+^ selectivity. The location of binding site 2 is thought to be at the S6 of all four domains and to overlap with the LA binding site or at least allosterically hinder LA binding.

**Figure 2:**
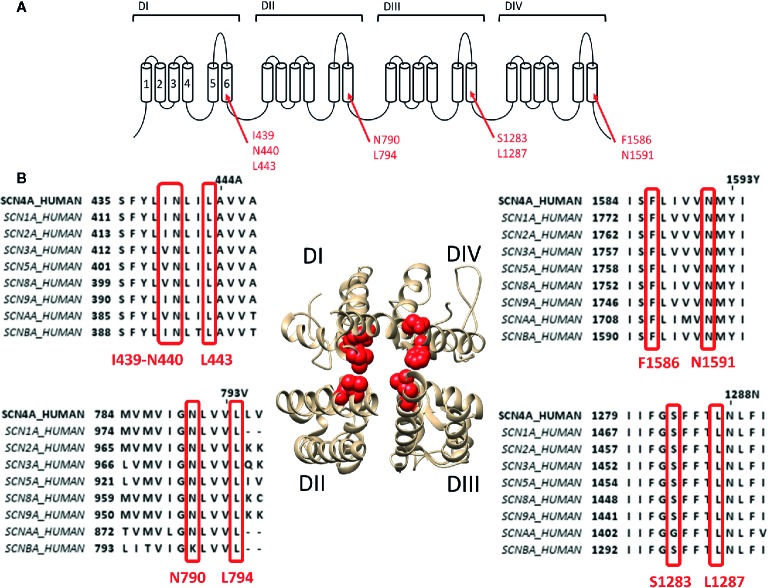
Location of binding site 2 within the Na_v_1.4 channel. **(A)** Schematic visualisation of the Na_v_ ion channel structure which is constructed out of four domains (DI to DIV) each consisting out of six transmembrane segments (S1–S6). The red arrows indicate the probable residues responsible for constructing the site 2 receptor, residues are noted in their Na_v_1.4 annotation. **(B)** The bottom view of a 3D structure of the Na_v_1.4 channel with in red marking the residues constructing the binding site 2. Only the S5–S6 segments of each domain is shown for a clear view on the inner pore binding site. Around the 3D structure are the alignments of the known human sodium channels with the Nav1.4 channel (SCN4A) as reference, all positioned at the corresponding domain of the 3D structure. Marked in red are the residues which are necessary for site 2 toxin binding. While some variability is observed between some isoform, most of the residues are well conserved. Amino acid sequence alignment and 3D structure are visualized using Jalview and chimera software, respectively (Pettersen et al., 2004; Waterhouse et al., 2009).

To describe neurotoxin site 2 in more detail, we focus on the most potent site 2 toxin reported to date, batrachotoxin (BTX) a steroidal alkaloid indirectly produced by the South-American poison dart frogs of the genus *Phyllobates* (Daly et al., 1965). Binding of BTX is highly irreversible and only possible when the channel is in its activated open state (Huang et al., 1982; Dubois et al., 1983). BTX shifts the voltage dependence of channel activation by approximately −40 mV toward more hyperpolarized potentials (Linford et al., 1998), reducing the channel's ion conductance and selectivity. BTX also reduces the affinity for LA, which is thought to be caused by non-competitive antagonism (i.e., allosteric effect) because of overlapping binding sites as BTX and LA share the binding residue F1586 (Linford et al., 1998). Residue mutations affecting BTX in Na_v_1.4 were first described in DI-S6 and DIV-S6: I439K, N440K, L443K, F1586K, and N1591K, respectively (Wang and Wang, 1998; Wang and Wang, 1999). Afterwards the DIII-S6 residues Ser1283 and leu1287 were identified (Wang et al., 2000) and finally DII-S6 residues N790 and L794 (Wang et al., 2001). All these residues line the inside of the pore (**Figure 2**). Non-pore lining residues involved in BTX sensitivity locate at the putative hinge region of the channel gate, Gly1282 and Phe1284, respectively (Du et al., 2011). While Phe1284 is important for the stabilisation of the ammonium group of BTX, Gly1282 is not a direct binding receptor for BTX and mutations of this residue affect binding allosterically by changing the channels gating properties. Modeling studies suggest that BTX binds to the pore but does not completely prevent ion conduction because of its “horseshoe-like” structure (Du et al., 2011). Only upon mutation of DII-S6 N790 BTX becomes a full blocker (Wang et al., 2007).

### Binding Site 5 Locates Between DIS6 and DIVS5

**Figure 3:**
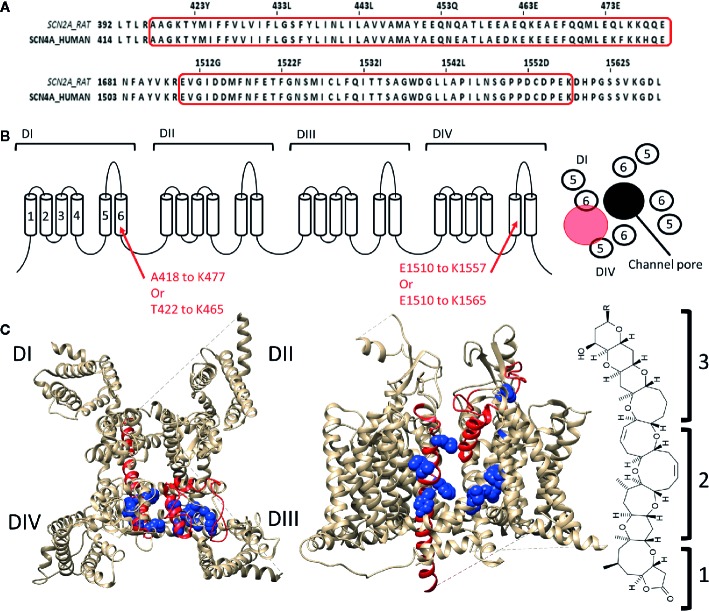
Location of binding site 5 in Na_v_1.4. **(A)** Alignment of the rat brain IIa sodium channel (SCN2A) with the human Na_v_1.4 (SCN4A) as reference. Marked in red are the regions where brevetoxin could bind. **(B)** Schematic topology of the Na_v_1.4 channel with the residues of DIS6 and DIVS5 that form the binding site listed below. On the right a schematic representation of the PD (top view) with the ion permeation pore represented in black and the location of binding site 5 indicated by the red circle. **(C)** 3D structures of the Na_v_1.4 channel seen from the top (left) and side (center, between DIII and DIV) are represented with in red the regions which are, using radioactive labelling studies, probably the location for brevetoxin binding. In blue residues are shown which had an effect on brevetoxin binding. With for DI M424, V429, I430, G434 decreasing affinity and F436, Y437 increasing affinity when mutated to alanine. In case of DIV mutation of I1485, G1486, L1488, L1489 lead to decrease and L1491, V1492, G1500, Y1506 to increase of affinity when mutated (Nav1.4 numbering) (Konoki et al., 2019). A cleft can be observed in between DIS6 and DIVS5, at the side view, where the toxin could bind. This cleft is also located around fenestration four of the ion channel. At the left the structure of brevetoxin with the head region (1) attached with a linker (2) to the tail structure (3). The structure is orientated as it would bind at the channel compared to the side view. Amino acid sequence alignment and 3D structures are visualized using Jalview and chimera software, respectively (Pettersen et al., 2004; Waterhouse et al., 2009).

Some of the toxins that bind to site 5 of Na_v_ channels are brevetoxins (Catterall and Gainer, 1985; Poli et al., 1986) and ciguatoxins (Murata et al., 1990; Lewis et al., 1998), both originate from marine dinoflagellates (*Gambierdiscus toxicus*) and are structurally comparable. Brevetoxins are produced by unarmoured marine dinoflagellates (e.g., Karenia brevis, Gymnodinium breve, or Ptychodiscus brevis). Ingestion of these toxins can lead to poisoning and death of marine animals and cause the disease neurotoxic shellfish poisoning in humans (Baden, 1983; Flewelling et al., 2005). Eleven different brevetoxins have been discovered today with brevetoxin A (PbTX1, CAS No.:98112-41-5) and brevetoxin B (PbTX2 and 3, CAS No.:79580-28-2 and 85079-48-7) being the most investigated ones. These toxins are about 30Å in length, 6Å in width, and 6Å high. They are composed of four recognizable parts: a lactone ring (the head) linked with a linker to a multiple carbon ring (the tail) ending in a rest-group (Lin et al., 1981). While the head is responsible for the effect, without the linker and the tail the toxin cannot modulate the channel as it allows the head region to reach its binding site (Rein et al., 1994). PbTX binds to the Na_v_ channel at the DI-S6, DIV-S5, and DIV-S6 segments (Trainer et al., 1991; Trainer et al., 1994; Konoki et al., 2019). The region spans within DI-S6 from Ala418/Thr422 to Lys465/Lys477 and within DIV-S5 from Glu1510 to lys1557 with possible extension to Lys1565 (**Figure 3**). The tail of the molecule will be situated at the S5–S6 extracellular loop while the head is able to reach, due to the long linker and tail region, the inactivation gate at the intracellular side of the channel (Trainer et al., 1991; Trainer et al., 1994). Interaction of the head with the inactivation gate reduces channel inactivation, leading to a persistent sodium current (Sheridan and Adler, 1989; Schreibmayer and Jeglitsch, 1992).

Like brevetoxins, ciguatoxins (CTX) bind at site 5 and their effects are consequently similar. Ciguatoxins are classified based on their geographic origin, with P-CTX (Murata et al., 1990) and C-CTX (Lewis et al., 1998) standing for Pacific and Caribbean, respectively. In all Na_v_ channel isoforms P-CTX1 (CAS No.:11050 21 8), the most potent CTX discovered to date, induces a hyperpolarizing shift in the voltage dependence of channel activation (Bidard et al., 1984; Benoit et al., 1986; Strachan et al., 1999) concomitantly with a shift in the voltage dependence of inactivation in some (Inserra et al., 2017). As CTX and brevetoxin share the same binding site, they are logically going into competition with each other (Lombet et al., 1987). Gambierol (CAS No.: 146763-62-4, origin *Gambierdiscus Toxicus*) has a similar structure as brevetoxins and CTXs, being a lipophilic multi-ring polyether toxin, but has no effect on the sodium currents (Lepage et al., 2007). However, when administered simultaneously it decreases the effect of CTX and brevetoxin, suggesting that gambierol acts as a competitive antagonist that binds to site 5 or at least exerts a negative allosteric effect.

## Voltage-Gated Potassium Channels

The K_v_ channel family is impressively large compared to this of Na_v_ channels, due to an extensive library of genes encoding different α-subunits that in some subfamilies can “mix-and-match” to form functional K_v_ channels. Additionally, alternative splicing, RNA editing, and post-translational modification further expand on the K_v_ channel family (Jan and Jan, 2012). This explains why the binding sites of the much larger K_v_ channel family are less well categorized than those of the Na_v_ channel family. The next part will highlight different binding sites and pore wall fenestrations within several, but not all, members of the K_v_ channel family. After a short overview of the well-documented extracellular and intracellular exposed “hydrophilic” binding sites, we will discuss in detail the lesser-known lipid embedded “hydrophobic” binding site(s) in different K_v_ channels.

### Extracellular and Intracellular Exposed “Hydrophilic” Binding Sites

The well-documented K_v_ channel binding sites can be topologically located on the intracellular or extracellular side of the channels. Extracellularly the most well-known conserved binding sites are those of the pore blockers and VSD targeting gating modifiers, while intracellularly the inner pore block is the most notable one (Wulff et al., 2009). Certain toxins from a variety of venomous animals target the extracellular binding sites. For example, scorpion toxins like charybdotoxin (CTX, CAS No.: 95751-30-7) and agitoxin (AgTx, CAS No.: 168147-41-9) target the water enveloped extracellular mouth of K_v_ channels, thereby physically occluding the permeation pore (Eriksson and Roux, 2002; Banerjee et al., 2013). Other scorpion toxins, like ergotoxin (ErgTx1, CAS No.: 8006-25-5) and BeKm-1 (CAS No.: 524962-01-4), cause an incomplete block of K_v_ current. This is because they only partially occlude the permeation pathway, which is known as “turret-block” (Xu et al., 2003; Zhang et al., 2003; Hill et al., 2007). Gating modifier toxins, like tarantula toxins (Hanatoxin, SGTx1, and VSTx1), bind to the paddle motif (S3b helices, S3–S4 linkers, and S4 helices) at the extracellular protein-lipid interface of the VSD. This binding site cannot be characterized as strictly hydrophilic as the hydrophobic residues of these amphipathic peptide toxins allow it to partially partition into the membrane, and interact with the VSD (Lee et al., 2004; Jung et al., 2005; Alabi et al., 2007; Swartz, 2007; Jung et al., 2010; Mihailescu et al., 2014). Intracellularly, compounds can bind in the inner cavity, either physically blocking ion permeation (e.g., quaternary ammonium ions) or allosterically modulating the gating machinery of K_v_ channels (e.g., 4-aminopyridine, CAS No.: 504-24-5) (Armstrong, 1971; Armstrong and Loboda, 2001; Del Camino et al., 2005). Also, the quaternary ammonium ion TEA can occlude the external K_v_ pore, similar to the scorpion toxins mentioned above (Luzhkov and Aqvist, 2001). Apart from the gating modifier binding site, which is partially enveloped by water, the binding sites mentioned here are exposed to an aqueous environment. Hence, referring them here as “hydrophilic” binding sites.

### Lipid Embedded “Hydrophobic” Binding Site(s) and Pore Wall Fenestrations in the *Shaker*-Type K_v_ Family, K_v_7 Family, and K_v_10.1/K_v_11.1 Channel

**Figure 4:**
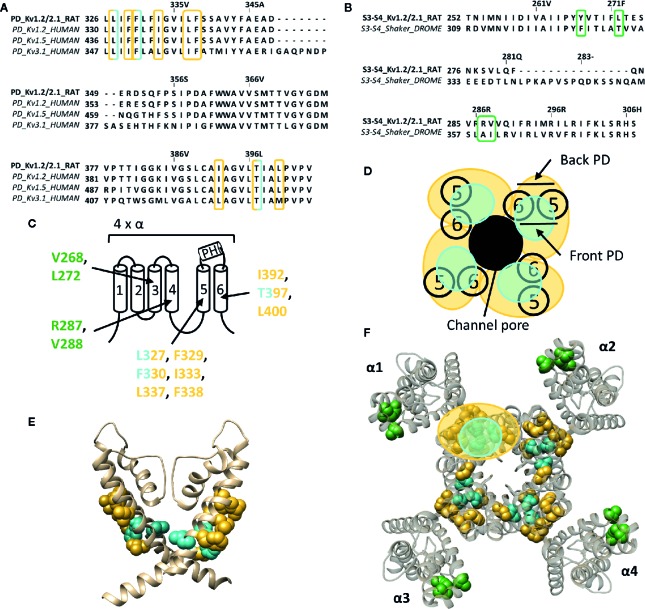
Lipid-exposed binding sites within the crystal structure of a K_v_1.2–K_v_2.1 paddle chimera channel (PDB: 2R9R). **(A)** Sequence alignment of part of the PD (S5-S6 segment) of K_v_1.2-2.1, K_v_1.2, K_v_1.5, and K_v_3.1, with K_v_1.2-2.1 as reference. Residues important for gambierol and psora-4 action are highlighted in blue and yellow respectively. **(B)** Sequence alignment of the S3–S4 segments of K_v_1.2–2.1 and *shaker*, with K_v_1.2–2.1 as reference. Highlighted in green are the residues important for PUFA action. **(C)** Schematic visualization of one K_v_ channel α-subunit consisting out of six transmembrane segments (1–6) and a pore helix (PH). In blue the residues important for gambierol binding (L327, F330, and T397 according to Kv1.2–2.1 numbering) and in yellow those for psora-4 binding (L327, F329, F330, I333, L337, F338, I392, T397, and L400). Residues important for PUFA interaction are shown in green (V268, R287, V288, and L272). **(D)** Schematic visualization of the pore domain of the K_v_1.2–K_v_2.1 channel. Four pore-forming domains tetramerize to form the channel pore. The blue and yellow circle highlights the proposed gambierol/psora-4 binding site regions on the front- and/or backside of the pore-forming domain, respectively. **(E)** Side view of the K_v_1.2–K_v_2.1 channel with the front and back subunit omitted for clarity. Residues involved in gambierol and psora-4 interaction are shown in blue (L327, F330, and T397) and yellow (I392, T397, and L400), respectively. The PUFA action site is visualized in green (V268, R287, V288, and L272). **(F)** Top view of the K_v_1.2–K_v_2.1 channel, with each α subunit named α1–α4. Residues involved in gambierol and psora-4 interaction are shown in blue (L327, F330, and T397) and yellow (I392, T397, and L400), respectively. In green the PUFA action site comprising residuesV268, R287, V288, and L272. K_v_1.2–K_v_2.1 crystal structure (PDB: 2R9R) (Long et al., 2007) was visualized with chimera software (Pettersen et al., 2004) and amino acid sequence alignment with Jalview (Waterhouse et al., 2009).

**Figure 5:**
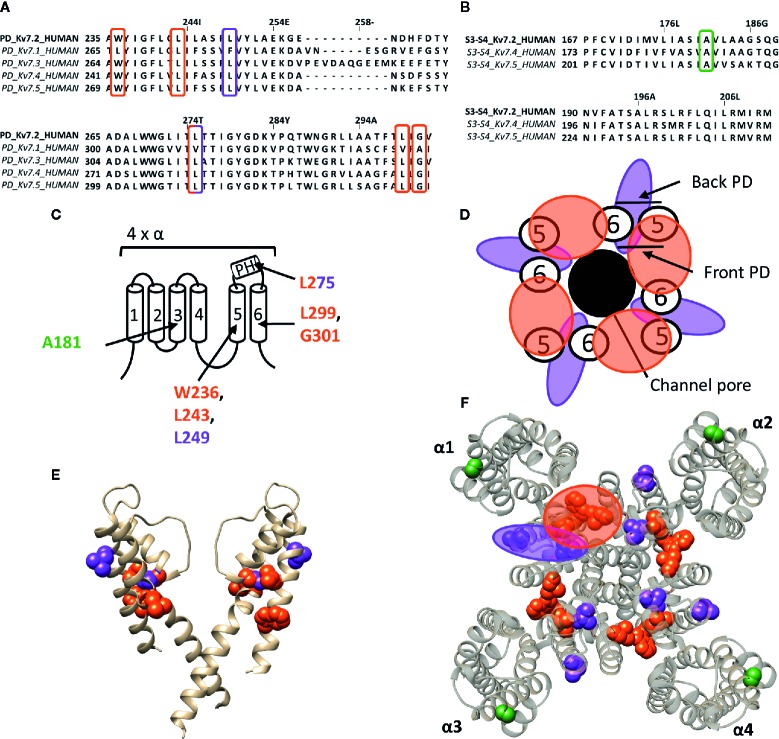
Lipid-exposed binding sites within a homology model of the K_v_7.2 channel based on the 3D structure of K_v_7.1 (PDB: 5VMS). **(A)** Sequence alignment of part of the PD of K_v_7.1–K_v_7.5, with K_v_7.2 residue numbering. Highlighted in red and purple are the residues important for RTG and zinc pyrithione action, respectively. **(B)** Sequence alignment of the S3-S4 segment of K_v_7.2, K_v_7.4, and K_v_7.5, with K_v_7.2 as reference. Highlighted in green is the residue important for ICA73 action. **(C)** Schematic visualization of one K_v_7 channel α-subunit consisting out of 6 transmembrane segments (1–6) and a pore helix (PH). Location of the residues involved in the interaction of K_v_7.2 with retigabine are represented in red. Represented in purple are residues important for zinc pyrithione action). The residue important for ICA73 interaction is shown in green. **(D)** Schematic visualization of the PD of K_v_7.2. The red and purple circle highlight the proposed retigabine/zinc pyrithione binding site on either the front- or back-side of the PD pore-forming domain, respectively. **(E)** Side view of K_v_7.2 with the front and back subunit omitted for clarity. Residues involved in retigabine and zinc pyrithione interaction are shown in red (W236, L243, L299, and G301) and purple (L249 and L275), respectively. In green the ICA73 action site is visualized (A181). **(F)** Top view of K_v_7.2, with each α subunit named α1–α4. Residues important for retigabine, zinc pyrithione, and ICA73 action are shown in red (W236, L243, L299, and G301), purple (L249 and L275), and green (A181), respectively. Shown K_v_7.2 structure is a homology model of the 3D structure of K_v_7.1 (PDB: 5VMS) (Sun and Mackinnon, 2017), generated with SWISS-MODEL (Waterhouse et al., 2018) and visualized using chimera software (Pettersen et al., 2004). The amino acid sequences are aligned using Jalview (Waterhouse et al., 2009).

**Figure 6:**
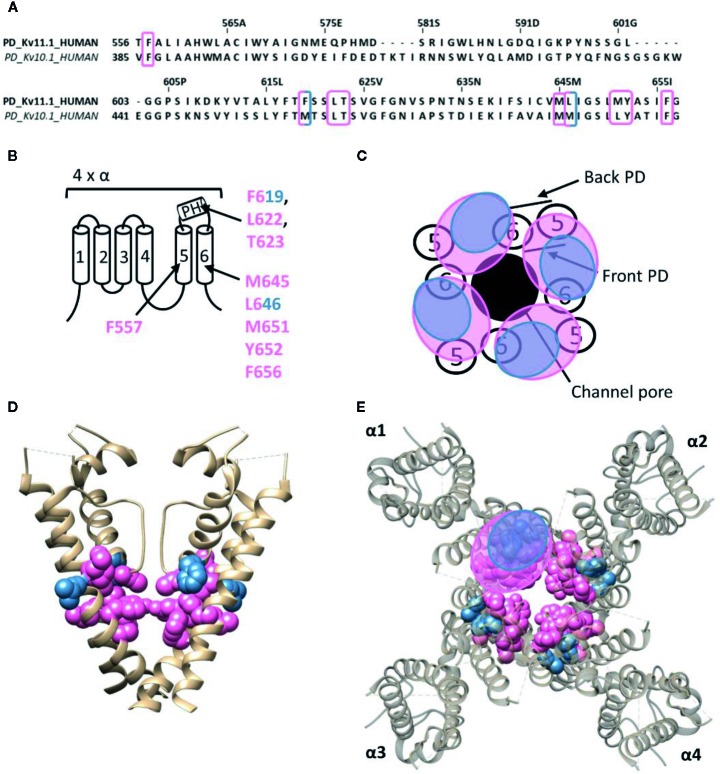
Lipid-exposed binding site(s) within the cryo-EM structure of the K_v_11.1 (hERG) channel (PDB: 5VA1). **(A)** Sequence alignment of part of the PD of K_v_11.1 and K_v_10.1, with K_v_11.1 residue numbering. Highlighted in pink and blue are the residues important for ICA74 and PD57 action, respectively. **(B)** Schematic visualization of one K_v_ channel α-subunit consisting out of six transmembrane segments (1–6) and a pore helix (PH). In blue the critical residues of PD57 (F619 and L646 according to Kv11.1 numbering) and in pink the residues lining the proposed hydrophobic ICA74 binding pocket (F619, F557, L622, T623, M645, L646, M651, Y652, and F656). **(C)** Schematic visualization of the pore domain of the K_v_11.1 channel. Four pore-forming domains tetramerize to form the channel pore. The blue and pink circle highlights the proposed PD57 and ICA74 binding site regions on the front- and/or backside of the pore-forming domain. **(D)** Side view of the K_v_11.1 channel with the front and back subunit omitted for clarity. Residues involved in PD57 and ICA74 interaction are shown in blue (F619 and L646) and pink (F619, F557, L622, T623, M645, L646, M651, Y652, and F656), respectively. The residues F619 and L646 are both critical residues for PD57 and ICA74, but are shown in blue. **(E)** Top view of the K_v_11.1 channel, with each α subunit named α1–α4. Residues involved in PD57 and ICA74 interaction are shown in blue (F619 and L646) and pink (F619, F557, L622, T623, M645, L646 M651, Y652, and F656), respectively. K_v_11.1 cryo-EM structure (PDB: 5VA1) (Wang and Mackinnon, 2017) was visualized with chimera software (Pettersen et al., 2004) and amino acid sequence alignment with Jalview (Waterhouse et al., 2009).

The next part reviews the potential lipophilic binding sites and pore wall fenestrations in different K_v_ channel types. To define the binding site(s) of certain compounds, the PD is divided into a “front side” and “back side”. Residues of the front side point towards the VSD of the same α-subunit, while residues of the “back side” point in the opposite direction, thus towards the VSD of a neighboring α-subunit (**Figures 4D**, **5D** and **6C**).

#### The *Shaker*-Type K_v_ Channel Family

Whereas gambierol does not modulate Nav channels, it is capable of inhibiting K_v_1 and K_v_3 channels (Nicholson and Lewis, 2006; Cuypers et al., 2008; Kopljar et al., 2009). Gambierol's inhibitory mechanism has been extensively studied, whereby a threonine on S6, T427 in K_v_3.1, is an important determinant (Kopljar et al., 2009). Substitution of the polar threonine by a hydrophobic valine abolishes the high gambierol affinity (Kopljar et al., 2009). Additional determinants are a leucine and phenylalanine on S5: L348 and F351 in K_v_3.1, respectively. K_v_1 channels possess a threonine residue equivalent to T427 (T401 in K_v_1.2), explaining their similar gambierol sensitivity (Cuypers et al., 2008; Kopljar et al., 2009; Martinez-Morales et al., 2016). These residues are mostly positioned on the front side of the PD (**Figure 4**).

On the other hand, psora-4 (CAS No.: 724709-68-6), a potent inhibitor of K_v_1.3, has been shown to predominantly bind to the back side of the PD (**Figure 4**) (Vennekamp et al., 2004; Marzian et al., 2013). A single psora-4 molecule acts as a central pore blocker of K_v_1 channels (K_v_1.1-K_v_1.5, and K_v_1.7), thereby preventing ions from permeating. However, four additional drug molecules can bind the lipid-exposed pocket on the back side of the PD, thereby causing the selectivity filter to narrow. Thus, the binding of five psora-4 molecules leads to a stable non-conducting state. The residues identified in K_v_1.5 as playing a key role in psora-4 action map on the back side of S5–S6 (**Figure 4**). Additionally, some residues in S4 and the S4–S5 linker also seem involved (Marzian et al., 2013).

The inhalation anaesthetic, sevoflurane, seems to bind within the central cavity and to a similar hydrophobic pocket as psora-4 (Stock et al., 2018). Apart from these binding regions, the polar lipophilic molecule has been shown to interact with the S4–S5 linker, pore helix, segment S6, and even the VSD, likewise to the many binding regions of sevoflurane on the Na_v_ channel (Barber et al., 2011; Barber et al., 2014). These sites are primarily dehydrated and lipid accessible, which is highly favourable for the polar lipophilic sevoflurane molecule (Stock et al., 2018). The specific residues involved in sevoflurane binding are not known, but one residue within the S4–S5 linker (G329 according to K_v_1.2 numbering) has been identified to play an important role (Liang et al., 2015).

PUFAs are charged lipophilic compounds that position at the lipid membrane interface, although in small quantities (Yazdi et al., 2016). Nonetheless, PUFAs may play an important role in the treatment of arrhythmias and epilepsy, due to their modulating effect on, but not limited to, voltage-gated ion channels (Lefevre and Aronson, 2000; Leaf, 2007; Boland and Drzewiecki, 2008; Borjesson and Elinder, 2011). The *Shaker* K_v_ channel has been extensively studied as one of the targets of PUFAs. They interact with the channel at several sites, but the major one seems located within the lipid-facing cleft between S3 and S4 (S3–S4 cleft) of the VSD (Borjesson and Elinder, 2011). This hydrophobic cleft is perfectly shaped to accommodate the lipophilic carbon tail of the PUFAs, causing the negatively charged carboxyl head group to be positioned close to the S4 segment. In this way, PUFAs electrostatically affect the VSD by trapping S4 toward the extracellular position, stabilizing the open state of *Shaker* K_v_ channels (Borjesson et al., 2008; Yazdi et al., 2016). Accordingly, a series of point mutations on the lipid facing side of S3-S4 (I325C, T329C, A359C, and I360C, *Shaker* numbering) had a significant impact on the PUFA-induced hyperpolarizing shift in the channel's voltage dependence of activation (**Figure 4**) (Borjesson and Elinder, 2011). Although the binding site for gating modifier toxins is in close proximity to that of PUFAs, the action sites do most likely not overlap as the residues important for PUFA action are more deeply embedded into the lipid bilayer (Borjesson and Elinder, 2011). Interestingly, dehydroabietic acid (DHAA) and some of its derivatives, the most potent being Wu32and Wu122, have a similar effect on *Shaker* K_v_ channels. The carboxyl group of DHAA is positioned at roughly the same site, namely the S3–S4 cleft (Ottosson et al., 2017). Wu32 possibly interacts with residues between the S2–S3 and/or S3–S4 cleft as five cysteine mutations in S3, which have been shown to alter its affinity and/or efficacy, point towards S2 (I320C and F324C, *shaker* numbering) and S4 (I318C, P322C, and T326C), respectively (Ottosson et al., 2017).

#### The K_v_7 Family

The well characterized retigabine (RTG, CAS No.: 150812-12-7) binding site is localized between the front side of one PD and the back side of an adjacent PD of K_v_7.2–K_v_7.5 (KCNQ2-5) channels (**Figure 5**) (Schenzer et al., 2005; Lange et al., 2009). These channels are predominantly expressed in neurons where they underlie the native M-current that plays a major role in regulating neuronal excitability (Wang et al., 1998; Kubisch et al., 1999; Schroeder et al., 2000). RTG amplifies the K_v_7.2–K_v_7.5 currents by stabilizing the open-channel conformation, by which it acts as a brake on neuronal excitability *in vivo* (Lange et al., 2009). Hence, the potential use of RTG and RTG-derived compounds as anticonvulsants (Rundfeldt, 1997; Wang et al., 2018). RTG binding has been attributed to several conserved residues on S5–S6, lining a hydrophobic pocket near the channel gate of K_v_7.2–K_v_7.5. According to K_v_7.2 numbering these residues are: W236, L243, L275, L299, and G301 (**Figure 5**). K_v_7.1 channels lack these amino acids (apart from L243), explaining their RTG-insensitivity (Schenzer et al., 2005; Lange et al., 2009). Because of the role of K_v_7 in diseases of neuronal hyperexcitability, the search for positive allosteric modulators such as RTG, for which the clinical use is currently discontinued (Wang et al., 2018), is pursued. For example, BMS-204352, ML-213, and the acrylamide compound (S)-1 act on the canonical RTG binding site (Bentzen et al., 2006; Kim et al., 2015; Wang et al., 2018).

The hydrophobic pocket of the RTG binding site also seems to be able to accommodate endogenous hydrophilic neurotransmitters like γ-aminobutyric acid (GABA), which directly activates K_v_7.3 and K_v_7.5 *via* W236. In contrast to RTG, GABA does not readily cross the plasma membrane to reach its site of action. Based on the K_v_1.2–K_v_2.1 paddle chimera structure the tryptophan also seems to be accessible from the extracellular side (Manville et al., 2018). It is possible that this accessibility is dependent of the state of the channel, such that in certain conformations the mostly hydrophobic binding pocket can be reached by hydrophilic compounds like GABA. Additionally, not all K_v_7 channel openers interact with residue W236. Zinc pyrithione for instance interacts with two residues at the back side of S5 and the pore helix (L249 and L275) (**Figure 5**) (Schenzer et al., 2005; Xiong et al., 2007; Lange et al., 2009).

ICA-compounds (ICAgen, Durham, NC, US), which are benzanilide K_v_7 channel openers, were developed as RTG alternatives (Mcnaughton-Smith et al., 2002). Of these, the most well-documented is ICA-27243 (ICA43), which has been shown to be more selective than RTG (Wickenden et al., 2008). The individual residues that determine ICA43 binding have not been identified, but the C-terminal end of S2 and the N-terminal part of S3 (S2–S3 cleft) are proposed to be involved (Padilla et al., 2009). Later, Wang AW, et al. continued the investigation of the mechanism of action of ICA-compounds on K_v_7 channels, but focussed on ICA-069673 (ICA73) (Wang et al., 2017). They identified two key residues in S3 of K_v_7.2: A181 and F168, respectively. Mutation of these residues did not affect RTG-mediated gating, but did alter the action of ICA73 (Wang et al., 2017). Furthermore, it has been shown that ICA43 and ICA73 are resistant to mutation of the RTG binding site, supporting that not all K_v_7 channel openers bind to the PD, but also can interact with a VSD site (Padilla et al., 2009; Wang et al., 2017). However, it remains debated whether residues A181 and F168 are involved in ICA binding directly or allosterically (Wang et al., 2018). In case these residues are binding ICA73, the position of residue A181 toward the lipid-exposed surface of the VSD suggests the presence of a drug binding site at the lipid-exposed cleft of S2–S3 and/or S3–S4 in K_v_7 channels, similar to the interaction of Wu32 with *Shaker* K_v_ channels (**Figure 5**).

PUFAs have also been described to electrostatically affect K_v_7.1 channels, resulting in a negative shift of the conductance-voltage curve. This modulation of K_v_7.1 channels by PUFAs is similar to what has been described for *shaker* K_v_ channels (Borjesson et al., 2008; Borjesson and Elinder, 2011). Hence, the binding site and mechanism of action of PUFAs are most likely similar for both channels (Liin et al., 2015).

#### The hERG (K_v_11.1) and EAG (K_v_10.1) Channel

The human *ether-a-go-go* related gene (hERG) type 1 encodes for the K_v_11.1 channel, which functions as the rapid component of the delayed rectifier K^+^ current contributing to the repolarization of cardiac action potentials (Vandenberg et al., 2012). Alteration of the native functioning of K_v_11.1 channels, either genetically or pharmacologically, can disrupt this repolarization, leading to various cardiac rhythm disorders (Thomas et al., 2006). Furthermore, the role of K_v_11.1 is not limited to the heart, as it also seems to play a role in the central nervous system, digestive, secretory, and reproductive system, and even cancer (Babcock and Li, 2013). Hence, it has become common practice to screen compounds on hERG channel activity during the early stages of drug development, as unintentional side-effects may lead to disease and sudden-death (Mitcheson et al., 2000; Thomas et al., 2006; Babcock and Li, 2013). Most hERG inhibitors interact with residues inside the channel's permeation pathway, either located on the pore helix (T623,S624, and V625, according to hERG numbering) or on segment S6 (G648, Y652, and F656) (Lees-Miller et al., 2000; Mitcheson et al., 2000; Kamiya et al., 2001; Sanchez-Chapula et al., 2002; Sanchez-Chapula et al., 2003; Fernandez et al., 2004; Kamiya et al., 2008). On the other hand, some small molecule hERG activators have been discovered who deviate from this binding site, like ICA-105574 (ICA74) and PD-118057 (PD57). The most critical binding determinants for ICA74 are F557 and L622 (Garg et al., 2011), which topologically would situate the binding site at the front side of the PD (**Figure 6**). Apart from these critical binding determinants several other residues have been proposed to line the hydrophobic ICA binding pocket of K_v_11.1 (F619, T623, M645, L646, M651, Y652, and F656), situating the ICA74 binding site between the front side of one α-subunit and the back side of an adjacent α-subunit (Garg et al., 2013). PD57 seems to bind the same hydrophobic pocket, with key residues being L646 on segment S6 and F619 on the pore helix of an adjacent subunit (**Figure 6**) (Perry et al., 2009).

Interestingly, ICA74 binds to a similar hydrophobic pocket in the related ether-a-go-go (EAG) type 1 channel (K_v_10.1), although eliciting an opposite effect as in K_v_11.1. ICA74 inhibits K_v_10.1 currents by enhancing channel inactivation (Garg et al., 2012). The key residues for ICA74 binding in K_v_11.1 (F557 and L622) are indeed conserved in K_v_10.1 (F359 and L434). Furthermore, only three residues (F619, L646, and M651) of the proposed ICA binding pocket in K_v_11.1 appeared not to be conserved in the K_v_10.1 channel. This strongly suggests that ICA74 binds to the same hydrophobic site in K_v_10.1 and K_v_11.1 channels (Garg et al., 2013).

#### Lateral Pore Wall Fenestrations

The reports of compounds that can access the inner cavity from lipid exposed side-pocket through lateral pore wall fenestrations in Kv channels is still very limited. In K_v_7.1 a pore wall fenestration is formed upon interaction with the β-subunit KCNE1 such that adamantane compounds, AC-1 (CAS No.: 878489-28-2) and its analogs (ACs), can reach their binding site (Jaraskova et al., 2005; Wrobel et al., 2016). Interestingly, AC-1 does not affect currents generated by homomeric K_v_7.1, channels, nor K_v_7.1 co-expressed with other KCNE isoforms (KCNE2-5). Thus, the “β-subunit-induced fenestrations” seem to be required for AC binding. Within these fenestrations many residues have been identified as important for AC-1 activity (V334, F335, I337, F340, and A344, according K_v_7.1 numbering), but its position relative to the central cavity and lipophilic side-pocket could not be elucidated (Wrobel et al., 2016).

In K_v_11.1, a lateral pore wall fenestration is possibly formed upon mutation of residue F557 to leucine (F557L), explaining the decrease in current inhibition of six known hERG blockers (dofetilide, haloperidol, terfenadine, astemizole, cisapride, and amiodarone) (Saxena et al., 2016). For K_v_1.5 it has been proposed that psora-4 molecules can move in the I502A mutant between the central cavity and the lipophilic side-pockets through fenestration between segments S5–S6 (Marzian et al., 2013).Hence, the presence of pore wall fenestrations has thus far only been observed upon β-subunit interaction with K_v_7.1 and to be induced by mutations in K_v_1.5 and K_v_11.1 (Marzian et al., 2013; Saxena et al., 2016; Wrobel et al., 2016). Although the presence of fenestrations has not been reported yet for wild-type channels, the likelihood that some K_v_ channel types express lateral pore wall fenestrations increases. If present, these fenestrations may be similar to those characterized in Na_v_ channels, which allow LA's to pass between the central cavity and lipophilic side-pockets (Payandeh et al., 2011; Mccusker et al., 2012; Payandeh et al., 2012; Zhang et al., 2012; Kaczmarski and Corry, 2014; Wrobel et al., 2016).

## Unifying the Lipid Exposed/Accessible Drug/Toxin Binding Sites of K_v_ and Na_v_ Channels

**Figure 7:**
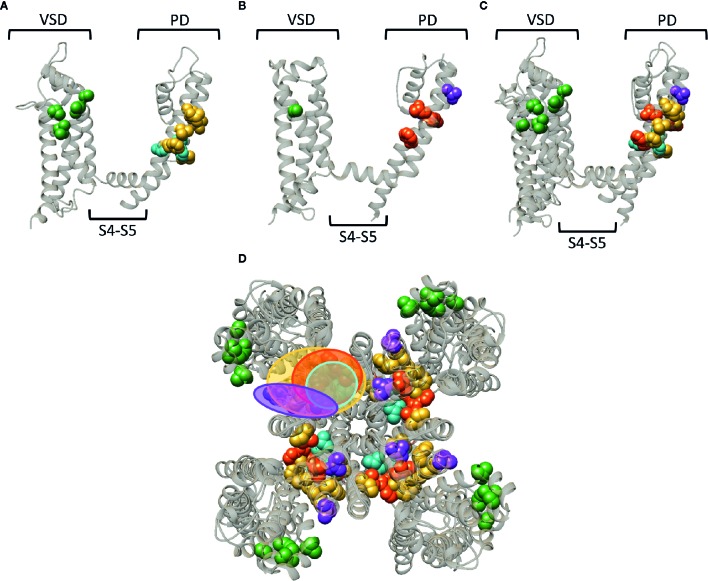
Superposition of the lipid-exposed binding sites within the K_v_1.2–2.1 paddle chimera channel (PDB: 2R9R) and K_v_7.2. **(A)** Side view of one α-subunit of the K_v_1.2–2.1 channel with the residues important for gambierol, psora-4, and PUFA action represented by spheres and highlighted in blue, yellow, and green, respectively. The VSD, PD, and S4–S5 linker are also indicated. **(B)** Side view of one K_v_7.2 α-subunit with the residues important for retigabine, zinc pyrithione, and ICA73 action represented by red, purple, and green spheres, respectively. **(C)** Superposition of the structures shown in panel **(A, B)**. **(D)** Superposition of the K_v_1.2–2.1 and K_v_7.2 channel structure shown as top view. The red and blue circle highlight the proposed retigabine/gambierol binding site on the “front side” of the PD, while the binding sites for zinc pyrithione and psora-4 on the “back side” of the PD are highlighted with the purple and yellow circles, respectively. Structures are visualized using chimera software (Pettersen et al., 2004).

Whereas a classification exists for the different drug/toxin binding sites in Na_v_ channels, such a categorization is currently lacking for the Kv channel family. In this review we take a first step and describe the hydrophobic binding sites reported in different K_v_ channel families. A compound like gambierol (**Figures 4** and **7**) has been shown to mostly bind to the front side of the PD, while RTG (**Figures 5** and **7**) PD57, and ICA74 (**Figure 6**) also interact with the back side of the PD of an adjacent α-subunit. When mapping all the sites it appears that gambierol, ICA74, RTG, and PD57 bind to an analogues binding site present in different K_v_ channel types (**Figure 7**) (Schenzer et al., 2005; Kopljar et al., 2009; Lange et al., 2009; Perry et al., 2009; Garg et al., 2011; Martinez-Morales et al., 2016). This indicates that an analogues lipophilic binding site is conserved between the different K_v_ channel types.

Zinc pyrithione, on the other hand, seems to solely bind to the back side of the PD, implying that the front and back side of the PD could serve as distinct binding sites (**Figures 5** and **7**) (Xiong et al., 2007). Psora-4 and sevoflurane are less specific regarding their binding site, as they both bind to the front and back side of the PD, among others (Marzian et al., 2013; Liang et al., 2015; Stock et al., 2018). Although certain compounds solely bind to the front or back side of the PD it seems that all residues point toward a similar lipophilic region, leading to the speculation that these seemingly distinct binding sites may converge to just one conserved lipophilic binding region in K_v_ channels. This binding site is then most likely similar to neurotoxin site 5 in Na_v_ channels (**Figure 3**) (Catterall and Risk, 1981; Cestele and Catterall, 2000), located between DIS6-DIVS5 (**Figures 3**, **4**, **5**) (Konoki et al., 2019). The idea that these binding sites are orthologous equivalents is because gambierol presumably binds to site 5 in Na_v_ channels (Lepage et al., 2007). In the case of K_v_ channels four such binding sites are present due to its tetrameric nature, as opposed to Na_v_ channels who only have one neurotoxin site 5 (Schenzer et al., 2005; Kopljar et al., 2009; Lange et al., 2009; Marzian et al., 2013; Stock et al., 2018).

Another lipid facing binding site is located on the VSD, in particular the cleft between segments S2–S3 and S3–S4. PUFAs, DHAA and its derivatives, and ICA-compounds allegedly bind to these clefts in *Shaker* and K_v_7 channels, respectively (**Figures 4D**, **5D**, and **7**) (Padilla et al., 2009; Borjesson and Elinder, 2011; Ottosson et al., 2017; Wang et al., 2017). This leads to the assumption that also this lipophilic binding site is conserved between different K_v_ channel types. Additionally, certain compounds of Na_v_ (LAs and sevoflurane) and K_v_ (AC-1, psora-4, and several hERG blockers) channels have been proposed to use hydrophobic lateral pore wall fenestrations to reach their binding sites. The location of these fenestrations in Na_v_ channels are situated between DI–DII and DIII–DIV (**Figure 1**), while in K_v_ channels they are most likely present between segments S5–S6, allowing lipid soluble compounds to reach their binding site even when the channel is in its closed state (Payandeh et al., 2011; Mccusker et al., 2012; Payandeh et al., 2012; Zhang et al., 2012; Marzian et al., 2013; Barber et al., 2014; Kaczmarski and Corry, 2014; Saxena et al., 2016; Wrobel et al., 2016).

Of note, the residues reported to affect drug/toxin affinity were in this review mapped on available 3D structures that are snapshots of the channel in a certain state, which should not be the high affinity state for the respective drug/toxin. As mentioned, several drugs/toxins are state dependent and bind with highest affinity to a certain conformation of the channel (e.g., the closed or open channel configuration). Consequently, the residues reported to be important for drug/toxin effect might orient differently when the conformation of the channel changes. Thus, when the channel is in its high affinity drug/toxin state, the orientation of the residues might be slightly different resulting in possibly broader binding regions than highlighted in the figures. Furthermore, several of the residues reported to be important for drug/toxin effect are likely not the binding partners of the drugs/toxins but alter affinity in an allosteric way. Nonetheless, there seem to be three distinct lipid-exposed binding sites preserved in K_v_ channels: the front and back side of the PD, and S2–S3/S3–S4 clefts. Future experiments will determine if the front and back PD binding sites are two distinct entities or if they converge to just one larger lipophilic binding site region.

## Author Contributions

All authors contributed in writing this review.

## Funding

This review was supported by a grant of the FWO (Fonds voor Wetenschappelijk onderzoek) G0C6220N (DJS) and by a GOA grant of the University of Antwerp.

## Conflict of Interest

The authors declare that the research was conducted in the absence of any commercial or financial relationships that could be construed as a potential conflict of interest.
